# Genetic analysis of lifetime productivity traits in goats

**DOI:** 10.5194/aab-64-293-2021

**Published:** 2021-07-06

**Authors:** Marie-Rosa Wolber, Henning Hamann, Pera Herold

**Affiliations:** 1 Institute of Agricultural Sciences in the Tropics, University of Hohenheim, Garbenstr. 17, 70599 Stuttgart, Germany; 2 State Agency for Spatial Information and Rural Development Baden-Württemberg, Stuttgarter Str. 161, 70806 Kornwestheim, Germany

## Abstract

As part of the development of a breeding programme for dairy goats to support sustainable production in organic farming, the overall aim of the present study was to identify traits that can be used as selection criteria for lifetime productivity. The breeding goal is high lifetime productivity with a good milk composition and a good level of robustness in the animals, especially within grazing systems. The lifetime productivity traits analysed in this study were the length of the animal's productive life (LPL), the lifetime efficiency (LEF), and the animal's milk yield efficiency with respect the total number of lactating days (EDM); the average fat and protein content over the animal's lifetime, the fat-to-protein ratio (FPR), and the urea content (UC) were also included as indirect health traits and potential indicators of robustness in dairy goats. The traits' influencing factors, phenotypic and genetic correlations, and heritability were examined. Furthermore, factors influencing milk yield in the first 120 d of lactation during the animal's first lactation were analysed. The aim of investigating milk yield during the first lactation was to consider a connection between early performance recoding in the life of an animal and LPL, LEF, and EDM. In total, lactation and pedigree data from 9192 dairy goats of the common German Fawn (GF) and German White (GW) dairy breeds were used. Prerequisites were that the investigated birth cohorts had to have definitively completed their lifetime production, and a high proportion of goats had to have completed extended lactation. The data analysis showed that breed did not influence milk yield. The age at first kidding, the average number of kids born during the animal's lifetime, and the lactation length did influence the milk yield. This applies to the milk yield during the first 120 d of the first lactation as well as over the lifetime of an animal. Considering the influencing factors, the results showed that LPL was genetically and positively correlated with LEF and EDM ($r_{g}=0.65\pm 0.06$ and 0.29±0.07 respectively). The heritability of LPL, LEF, and EDM was 0.22±0.02, 0.29±0.03, and 0.44±0.03 respectively. Regarding the lifetime milk composition, the heritability of protein and fat content, FPR, and UC was 0.63±0.02, 0.52±0.02, 0.32±0.03, and 0.47±0.04 respectively. The heritability regarding the milk yield during the first 120 d of the first lactation was 0.34±0.03. We found that the milk yield during the first 120 d of the first lactation showed a genetic correlation with LPL, LEF, and EDM of 0.30±0.08, 0.82±0.04, and 0.89±0.03 respectively. In summary, LPL, LEF, and EDM are suitable traits to indicate lifetime productivity in dairy goats. An additional indicator for lifetime productivity could be the milk yield during the first 120 d of the first lactation. Moreover, FPR and UC appear to be promising indicator traits for the health and robustness of dairy goats. The present study showed the importance of considering extended lactation in selective breeding programmes as well as the importance of modelling extended lactation in the breeding value estimation.

## 1 Introduction

Breeding structures for dairy goats in Germany are poorly developed (Zumbach and Peters, 2007), and artificial insemination and structured breeding programmes currently do not exist. Compared with other livestock species, goat breeding in Germany is in its very early stages, and goat breeding organizations in the federal states of Bavaria and Baden-Württemberg only started to intensify their goat breeding programmes a few years ago. Herold et al. (2018) provides a brief overview of the goat breeding framework as well as recent developments. Despite the small breeding population, a breeding value estimation for the main two dairy goat breeds, German Fawn (GF) and German White (GW), was implemented in 2014. It was started using the available data from milk recording. Dairy goat farms in Germany are mainly organic (Herold et al., 2007; Manek et al., 2017); therefore, any attempts to improve the breeding structure in dairy goats must take the conditions and limitations of organic farming systems into account. The breeding goal is high lifetime productivity with a good milk composition and a good level of robustness in the animals, especially within grazing systems (Herold, 2016).

Additionally, breeding programmes should focus more on the robustness of dairy goats. Robustness describes the ability of an animal to maintain (high) production and wellbeing while being affected by different stressors (e.g. Friggens et al., 2017; Llonch et al., 2020). Robustness is a combination of multiple, interacting components (Friggens et al., 2017). It is difficult to define traits that reflect the biological mechanisms of robustness and that fulfil the requirements of selection traits (heritable, biologically meaningful, and repeatable). One possibility to describe robustness is to use biomarkers as indicators. In the present study, the fat-to-protein ratio (FPR) and the urea content (UC) were used in such a manner. The FPR and UC are possible indicators of the animal's energy status or metabolic robustness (Buttchereit et al., 2011; König and May, 2019; Mäntysaari et al., 2019). Additionally, according to Friggens et al. (2017), longevity and lifetime efficiency are good measures of robustness in the same or similar environments.

Thus, the overall aim of the present study was to identify traits measured in regular milk recording that are suitable as selection criteria for lifetime productivity and robustness. For this reason, the traits' influencing factors, phenotypic and genetic correlations, and heritability were examined. Overall, the lifetime productivity traits investigated were the length of the animal's productive life (LPL), the lifetime efficiency (LEF), the animal's milk yield efficiency with respect the total number of lactating days (EDM), and the average fat and protein content over the animal's lifetime. Furthermore, factors influencing milk yield during the first 120 d of lactation during the animal's first lactation were analysed to consider a connection between the beginning of lactation and lifetime efficiency. In addition, an analysis was undertaken in order to establish if FPR and UC data from milk recording are suitable for use as robustness indicator traits.

## 2 Materials and methods

### 2.1 Dairy goat breeding and husbandry in southern Germany

According to Manek et al. (2017), most of the 35 000 dairy goats in Germany are located in Bavaria, a federal state of Southern Germany. In 2020, 72 farms with a total of 5782 dairy goats participated in milk performance testing (LKV Bayern, 2020). At the same time, 2555 German Fawn goats from 41 farms and 1337 German White goats from 22 farms were registered in the herdbook of the Bavarian breeders association (Mendel, unpublished data). Dairy goat keeping as a fulltime farm activity and main source of income is relatively new and has developed over the last 25 years. The majority (81 %) of the goat farms in southern Germany (Baden-Württemberg and Bavaria) are organic operations (Kern, 2019).

With an increasing number of dairy goat farms and an increasing number of dairy plants processing goat milk, the number of days that an animal is kept lactating (and is milked) has increased in recent years. If milking continues for more than 1 year or even several years, lactation is characterized as “extended” (Moog et al., 2012). Extended lactation can occur before actual lactation, separated by a kidding. In addition, actual lactation can result in continuous milking and, therewith, in extended lactation. Major reasons for extended lactation are (1) that dairy plants demand a continuous supply of milk, and they pay better prices in autumn, and (2) there is no market for surplus kids (Ringdorfer, 2009). Therefore, extended lactation for several years can be an economically interesting option for dairy goat farms (Schuiling, 2007).

To date, lifetime productivity traits have not been investigated in German dairy goats. Working on lifetime productivity in an actual population means working with censored records. Therefore, the present study, as a pilot study for lifetime productivity traits in the German dairy goat population, deals with data from goats that have already ended their productive lifetime. The aim of this work was to estimate reliable variance components based on historical records. Unfortunately, this approach encountered two restrictions. The first restriction was that, due to the poorly developed breeding structures in the past, there was a high number of missing pedigrees in the records utilized. These data were available on paper, but they were not not available for data processing: unfortunately, there was neither the personnel nor the money available to digitize the data. The second restriction was the fact that extended lactation has become more common over the last 10 years. Data were restricted to records from the Bavarian goat herdbook because farmers in this federal state were the pioneers of extended milking in Germany (Herold et al., 2018). This guaranteed a preferably balanced data set of normal and extended lactation.

### 2.2 Animals and material

Data were provided by the Landeskuratorium der Erzeugerringe für tierische Veredelung in Bayern e.V. in Bavaria. This study included data from goats born between 1988 and 2006. Prematurely deceased animals were excluded from the data set. Data preparation and plausibility checks were carried out using SAS 9.4 (2017). In total, lactation data from 9192 dairy goats raised on 226 farms were examined. Overall, 145 farms kept German Fawn goats (GF; n = 3868), 14 farms kept German White goats (GW; n = 103), and 67 farms kept both breeds (n = 3860 for GF and n = 1361 for GW). Table 1 describes the data stratified by breed and farm size. The end of a goat's life was defined as the day it left the farm. If the animal did not leave the farm, either the last milk recording test day or the last recorded kidding was counted as the day of leaving. The first kidding age, as defined by Herold et al. (2018), was divided into two “classes of first kidding age” (Cfk): Cfk 1 was ≤ 620 d and Cfk 2 was ≥ 621 d. For the GF breed, 180 sires and 2305 dams were recorded; for the GW breed, 52 sires and 355 dams were recorded. As such, information about the sire was available for 720 animals, and information about the dam was available for 3786 GF animals. Among the GW population, there were 153 dairy goats with sire information and 526 dairy goats with dam information. The ancestry of many animals was unknown, which was mainly due to the poorly developed data-processing structures in goat breeding. Goat pedigrees used to be stored on paper, and goat ear tag numbers have only been recorded digitally since the launch of a central database in 2009. Moreover, as a result of the livestock traffic ordinance put in place around this time, each dairy goat is now identified digitally using an individual animal number. Not all pedigree data for dairy goats born before 2009 have been digitized, and some identification numbers were not clearly assignable. Table 2 shows the data structure of the investigated dairy goats. Targeted lifetime productivity traits were the length of the animal's productive life (LPL), the lifetime efficiency (LEF), the animal's milk yield efficiency with respect the total number of lactating days (EDM), and the average fat and protein content over the animal's lifetime. Further, milk yield during the first 120 d of lactation during the animal's first lactation was examined. The traits were defined as follows: LPL (in days) was the time period from first kidding to the last information available for the animal, LEF was lifetime milk yield (in kg per day), and EDM was the efficiency per day with respect to milk yield per day of lactation (in kg per day). LEF includes rearing and dry-off periods, because lifetime milk yield implies that the rearing phase is included. LPL includes the dry-off period. EDM describes the animal's milk yield efficiency with respect the total number of lactating days and contains neither the rearing nor dry-off periods. Due to the prerequisite that all investigated goats had completed their lifetime productivity, information was available on total production per goat and on the length of the lifetime of each goat.

**Table 1:** Number of dairy goats and number of farms by breed and farm size.

Farm size	GF${}^{*}$		GW${}^{*}$		Mixed GF and GW	
(No. goats per farm)	No. goats	No. farms	No. goats	No. farms	No. goats (GF, GW)	No. farms
1–10	374	97	52	13	56, 38	16
10–20	245	18	0	0	134, 90	15
21–50	493	14	0	0	77, 139	6
51–100	265	4	51	1	740, 251	15
101–250	1442	9	0	0	883, 782	10
251–600	1049	3	0	0	1970, 61	5
No. observations	3868	145	103	14	3860, 1361	67

${}^{*}$ GF and GW denote the German Fawn and German White breeds respectively.

LPL, LEF, and EDM were chosen due to the different considerations in relation to the rearing and dry-off periods. In addition to examining the milk yield and the protein and fat content during lactation, the fat-to-protein ratio (FPR; the quotient between fat and protein content as a percentage) and the urea content (UC; in mg/100 mL) in milk were examined as part of the milk recording. In the data evaluation, extended lactation was examined. Extended lactation is becoming increasingly common as a method of milking animals over a long period without requiring them to birth offspring (Moog et al., 2012; Sehested et al., 2019). This practice could be an influencing factor on the lifetime traits examined, which is why it has been taken into account in the present investigation. The standard lactation length is 240 d (Herold et al., 2018). In a “normal” goat's life, one 365 d cycle with an annual kidding consists of a maximum lactation period of 305 d days and a non-lactation period of 60 d. Thus, the gestation period comprises 150 lactating days and a non-lactation period. As such, lactation was considered to be extended if the animal was lactating for more than 305 d. For more information on extended lactation in goats, see Wolber et al. (2018, 2019). Goats in the present data set predominantly underwent extended lactation during their second lactation (Wolber et al., 2018). Some goats were milked longer than 720 d. In the present work, the days of extended lactation over an animal's lifetime were calculated pro rata with respect to the total lactation days. These proportions were grouped into four extended lactation (EL) classes: EL = 0 %, 0 < EL ≤ 50 %, 50 < EL < 100 %, and EL = 100 %. Lactation traits and the number of kids in a goat's lifetime are also shown in Table 2. Table 3 shows the milk yield in the first 120 d of lactation during the animal's first lactation, the first kidding age (in days), the number of kids, and the proportion of animals that underwent extended lactation during their first lactation.

**Table 2:** Data collected over a dairy goat's lifetime by breed.

Data structure	GF${}^{a}$ (n = 7728)		GW${}^{a}$ (n = 1464)	
	$\bar{X}$	SD	$\bar{X}$	SD
Length of productive life (LPL; in days)	1146.70	901.87	976.84	763.26
Lifetime efficiency (LEF; in kg)	1.06	0.55	0.98	0.53
Milk yield efficiency with respect the total number of lactating days (EDM; in kg)	2.38	0.79	2.52	0.83
Prot. cont. (%)	3.29	0.29	3.32	0.26
Fat cont. (%)	3.56	0.54	3.51	0.48
Fat-to-protein ratio (FPR)	1.08	0.15	1.06	0.12
Urea content (UC)${}^{b}$	47.15	8.98	49.42	7.71
No. kids per dairy goat within the animal's lifetime${}^{c}$	1.85	0.51	1.79	0.50
Proportion of extended lactation (%)${}^{d}$	24.74	34.86	24.14	35.57

${}^{a}$ GF and GW denote the German Fawn and German White breeds respectively. ${}^{b}$ Reduced number of data available: n = 4951 (GF) and n = 827 (GW). ${}^{c}$ Reduced number of data available: n = 7535 (GF) and n = 1375 (GW). ${}^{d}$ Days of extended lactation over an animal's lifetime were calculated pro rata with respect to the total lactation days, and the proportions were grouped into four extended lactation (EL) classes: EL = 0 %, 0 % < EL ≤ 50 %, 50 % < EL < 100 %, and EL = 100 %.

**Table 3:** Data structure of dairy goats during the first 120 d of the first lactation.

Data structure	GF${}^{a}$ (n = 7728)		GW${}^{a}$ (n = 1464)	
	$\bar{X}$	SD	$\bar{X}$	SD
Milk yield (kg)	281.49	109.06	299.25	107.05
First kidding age (in days)	517.59	210.29	608.95	265.05
No. of kids during first lactation${}^{b}$	1.72	0.62	1.67	0.59
Proportion of extended lactation during first lactation (%)${}^{c}$	18.0	39.0	19.0	40.0

${}^{a}$ GF and GW denote the German Fawn and German White breeds respectively. ${}^{b}$ Reduced number of data available: n = 7532 (GF) and n = 1375 (GW). ${}^{c}$ Days of extended lactation over an animal's lifetime were calculated pro rata with respect to the total lactation days, and the proportions were grouped into four extended lactation (EL) classes: EL = 0 %, E ≤ 50 %, EL < 100 %, and EL = 100 %

### 2.3 Statistical analysis – analysis of variance

An analysis of variance was carried out using the SAS 9.4 (2017) statistical program and the SAS MIXED procedure. This was to determine which factors influenced LPL, LEF, and EDM. A pairwise difference test (PDIFF) was used to investigate the significant differences in trait expression (p<0.05). The following model equation was used to analyse factors influencing the lifetime output:

1 $$\begin{array}{rl} Y_{ijklmn} & =\mu +B_{i}+{\mathrm{By}}_{j}+{\mathrm{Cfk}}_{k}+{\mathrm{Ls}}_{l}+{\mathrm{El}}_{m}\\ & +{\mathrm{farm}}_{n}+e_{ijklmn}. \end{array}$$

Here, $Y_{ijklmn}$ denotes the observed lifetime productivity traits (LPL, LEF, and EDM), the average fat and protein content over the animal's lifetime, and the FPR and UC indicator traits. $B_{i}$ is the fixed effect of the breed (i can be 1 or 2, where GF is denoted by 1, and GW is denoted by 2), ${\mathrm{By}}_{j}$ is the fixed effect of the birth year (j ranges from 1 to 19, denoting the years from 1988 to 2006 respectively), Cfk${}_{k}$ is the fixed effect of the class of the first kidding age (k can be 1 or 2, where Cfk ≤ 620 d is 1 and Cfk ≥ 621 d is 2), Ls${}_{l}$ is the fixed effect of litter size (lifetime average; l ranges from 1 to 3, where a litter size of 1 is denoted by 1, a litter size > 1 is denoted by 2, and an unknown litter size is denoted by 3), El${}_{m}$ is the fixed effect of extended lactation with respect to total days lactating (m ranges from 1 to 4, where EL = 0 % is denoted by 1, 0 % < EL ≤ 50 % is denoted by 2, 50 % < EL < 100 % is denoted by 3, and EL = 100 % is denoted by 4), farm${}_{n}$ is the random effect of the farm (n ranges from 1 to 226), and $e_{ijklmn}$ is the residual error. The following model equation was used to analyse factors influencing milk production in the first 120 d of lactation during the animal's first lactation:

2 $$\begin{array}{rl} Y_{ijklmn} & =\mu +B_{i}+{\mathrm{By}}_{j}+{\mathrm{Cfk}}_{k}+\mathrm{Ls}1_{l}+\mathrm{El}\_1_{m}\\ & +{\mathrm{farm}}_{n}+e_{ijklmn}. \end{array}$$

Here, $Y_{ijklmn}$ is the milk yield in the first 120 d during the animal's first lactation, $B_{i}$ is the fixed effect of the breed (i can be 1 or 2, where GF is denoted by 1, and GW is denoted by 2), By${}_{j}$ is the fixed effect of the birth year (j ranges from 1 to 19, denoting the years from 1988 to 2006 respectively), Cfk${}_{k}$ is a fixed effect of the class of first kidding age (k can be 1 or 2, where Cfk ≤ 620 d is 1 and Cfk ≥ 621 d is 2), Ls1${}_{l}$ is the fixed effect of litter size at first kidding (l ranges from 1 to 3, where a litter size of 1 is denoted by 1, a litter size > 1 is denoted by 2, and an unknown litter size is denoted by 3), El_1${}_{m}$ is the fixed effect of extended lactation from the first lactation (m can be 1 or 2, where standard lactation is 1 and extended lactation is 2), farm${}_{n}$ is the random effect of the farm (n ranges from 1 to 226), and $e_{ijklmn}$ is the residual error.

### 2.4 Statistical analysis – estimation of variance components

Genetic parameters were estimated by using an animal model and applying the restricted maximum likelihood (REML) approach in VCE 6 software (Groeneveld et al., 2010). As part of the REML process, only converged estimations with a status of one were used in the multivariate estimation runs. The following model equation was used to estimate the variance components:

3 $$\begin{array}{rl} Y_{ijklmno} & =\mu +B_{i}+{\mathrm{Cfk}}_{j}+{\mathrm{Ls}}_{k}+\mathrm{El}\_1_{l}+b\cdot \mathrm{Sel}\_{\mathrm{lif}}_{m}\\ & +{\mathrm{hby}}_{n}+a_{o}+e_{ijklmno}. \end{array}$$

Here, $Y_{ijklmno}$ denotes the observed lifetime productivity traits (LPL, LEF, and EDM), the average fat and protein content over the animal's lifetime, and the FPR and UC indicator traits. $B_{i}$ is the fixed effect of the breed (i can be 1 or 2, where GF is 1 and GW is 2), Cfk${}_{j}$ is a fixed effect of the class of the first kidding age (j can be 1 or 2, where Cfk ≤ 620 d is 1 and Cfk ≥ 621 d is 2), Ls${}_{k}$ is the fixed effect of litter size (lifetime average; l can be 1 or 2, where a litter size of 1 is 1 and litter size > 1 is 2), and El${}_{m}$ is the fixed effect of extended lactation with respect to total days lactating (m ranges from 1 to 4, where EL = 0 % is 1, 0 % < EL ≤ 50 % is 2, 50 % < EL < 100 % is 3, and EL = 100 % is 4). The linear regression includes the linear regression coefficient (b) and the covariable Sel_lif${}_{m}$ (proportion of days lactating that are classified as extended lactation). The random effects are hby${}_{n}$, which is the herd birth year (n = 1.085), and $a_{o}$, which is the additive genetic effect of the animal (n = 1–25, 450). The residual error is $e_{ijklmno}$.

The following model equation was used to estimate the variance components for milk yield in the first 120 d of the first lactation:

4 $$\begin{array}{rl} Y_{ijklmno} & =\mu +B_{i}+{\mathrm{Cfk}}_{j}+\mathrm{Ls}1_{k}+\mathrm{El}\_1_{l}+b\cdot \mathrm{Sel}\_{\mathrm{lif}}_{m}\\ & +{\mathrm{hky}}_{n}+a_{o}+e_{ijklmno}. \end{array}$$

Here, $Y_{ijklmno}$ is the milk yield in the first 120 d during the first lactation. $B_{i}$ is the fixed effect of the breed (i can be 1 or 2, where GF is 1 and GW is 2), Cfk${}_{j}$ is a fixed effect of the class of the first kidding age (j can be 1 or 2, where Cfk ≤ 620 d is 1 and Cfk ≥ 621 d is 2), Ls1${}_{k}$ is the fixed effect of litter size at first kidding (l can be 1 or 2, where a litter size of 1 is 1 and litter size > 1 is 2), El_1${}_{l}$ is the fixed effect of extended lactation from the first lactation (m can be 1 or 2, where standard lactation is 1 and extended lactation is 2). The linear regression includes the linear regression coefficient (b) and the covariable Sel_lif${}_{m}$ (proportion of days lactating that are classified as extended lactation). The random effects are hky_1${}_{m}$, which is the random effect of the herd kidding year of the first lactation (m = 948), and $a_{o}$, which is the additive genetic effect of the animal (n = 1–25, 450). The residual error is $e_{ijklmno}$.

## 3 Results

### 3.1 Influence on lifetime productivity traits

The results of the analysis of variance are summarized in Table 4. Breed had no significant effect on lifetime productivity traits. First kidding age showed significant effects on the LEF and EDM: when goats kidded for the first time at more than 620 d, the LEF decreased by 0.14 kg and the EDM increased by 0.06 kg. An average litter size larger than one had significantly positive effects on the LEF and EDM. Apart from the FPR, extended lactation had a significant effect on all traits studied. If less than 50 % of the days lactating during the animal's lifetime were extended lactation, the phenotypically highest LPL, LEF, and EDM could be achieved, with values of 1415.58 d, 1.30 kg, and 3.08 kg respectively. If an animal had no extended lactation, significantly lower LSM (least squares mean) values were observed for LPL, LEF, and EDM, with values of 435.37 d, 0.76 kg, and 2.90 kg respectively. Animals without extended lactation during their lifetime had the significantly lowest LPL and LEF, with values of 435.37 d and 0.76 kg. The EDM was not significantly different from animals with 100 % extended lactation (2.90 kg vs. 2.88 kg).

**Table 4:** LSM values during the dairy goats' lifetime.

Factor levels during	No. obs.	LPL		LEF		EDM		Prot. cont. (%).		Fat cont. (%)		FPR		UC${}^{*}$	
lifetime		LSM	SE	LSM	SE	LSM	SE	LSM	SE	LSM	SE	LSM	SE	LSM	SE
GF	7728	996.53${}^{a}$	34.45	1.10${}^{a}$	0.03	2.94${}^{a}$	0.06	3.29${}^{a}$	0.01	3.60${}^{a}$	0.03	1.09${}^{a}$	0.01	46.87${}^{a}$	0.88
GW	1464	962.32${}^{a}$	40.48	1.11${}^{a}$	0.03	2.98${}^{a}$	0.06	3.31${}^{b}$	0.01	3.56${}^{a}$	0.03	1.08${}^{b}$	0.01	47.47${}^{a}$	0.92
Birth year (1988–2006)	9192	${}^{***}$													
Cfk 1: ≤ 620 d	6578	970.38${}^{a}$	36.00	1.17${}^{a}$	0.03	2.93${}^{a}$	0.06	3.30${}^{a}$	0.01	3.57${}^{a}$	0.03	1.08${}^{a}$	0.01	47.25${}^{a}$	0.88
Cfk 2: ≥ 621 d	2614	988.48${}^{a}$	36.34	1.03${}^{b}$	0.03	2.99${}^{b}$	0.06	3.31${}^{a}$	0.01	3.59${}^{a}$	0.03	1.09${}^{a}$	0.01	47.09${}^{a}$	0.89
Litter size = 1	3336	877.99${}^{a}$	37.10	1.03${}^{a}$	0.03	2.77${}^{a}$	0.06	3.28${}^{a}$	0.01	3.58${}^{a}$	0.03	1.09${}^{a}$	0.01	47.09${}^{a}$	0.89
Litter size >1	5571	1308.97${}^{a}$	32.82	1.32${}^{b}$	0.03	2.96${}^{b}$	0.06	3.30${}^{b}$	0.01	3.56${}^{a}$	0.03	1.08${}^{b}$	0.01	47.06${}^{a}$	0.86
EL = 0 %	5587	435.37${}^{a}$	33.97	0.76${}^{a}$	0.03	2.90${}^{a}$	0.06	3.24${}^{a}$	0.01	3.52${}^{a}$	0.03	1.09${}^{a}$	0.01	46.75${}^{a}$	0.88
0 % < EL ≤ 50 %	1323	1416.58${}^{b}$	39.54	1.30${}^{b}$	0.03	3.08${}^{c}$	0.06	3.29${}^{b}$	0.01	3.56${}^{b}$	0.03	1.08${}^{a}$	0.01	47.32${}^{\mathrm{ab}}$	0.90
50 % < EL < 100 %	1496	1242.49${}^{c}$	39.15	1.25${}^{c}$	0.03	2.98${}^{b}$	0.06	3.32${}^{c}$	0.01	3.60${}^{c}$	0.03	1.09${}^{a}$	0.01	47.39${}^{b}$	0.90
EL = 100 %	786	823.28${}^{d}$	41.79	1.11${}^{d}$	0.03	2.88${}^{a}$	0.06	3.36${}^{d}$	0.01	3.64${}^{c}$	0.03	1.08${}^{a}$	0.01	47.21${}^{\mathrm{ab}}$	0.92
No. obs.		9192													

${}^{a,\;b,\;c,\;d}$ Indices within the traits show significant differences at the p<0.05 level. The lifetime productivity traits are as follows: length of productive life (LPL; in days), lifetime efficiency (LEF; in kg), milk yield efficiency with respect the total number of lactating days (EDM; in kg), protein content (prot. cont.; in %), fat content (fat cont.; in %), fat-to-protein ratio (FPR), and urea content in milk (UC; in mg/100 mL). The factor levels are as follows: class of first kidding age (Cfk) and proportion of extended lactation in the animals total lactation (EL). ${}^{*}$ Measurements taken over the time span from 1995 to 2006 (n = 5778). ${}^{***}$ p<0.05.

The milk components were affected differently, which is also reflected in the effects on FPR. The average protein content over the animals' lifetime (in %) was influenced by the litter size class and by the intensity of extended lactation. An average litter size larger than one had a significant positive effect on the protein content (in %) of the milk. Goats with with 100 % extended lactation showed the highest protein content of 3.36 %. Fat content (in %) was also significantly affected by the intensity of extended lactation. As for protein, goats with 100 % extended lactation had the highest fat content of 3.45 %. The FPR during the animals' lifetime was significantly affected by both breed and the litter size class. GF had a higher FPR than GW (1.09 vs. 1.08), and goats with an average litter size of one showed a higher FPR than goats with an average litter size larger than one (1.09 vs. 1.08). The UC during the animals' lifetime was significantly affected by the share of extended lactation. Goats with no extended lactation had a lower UC than goats with a share of extended lactation between >50 and <100 % (46.75 vs. 47.39).

### 3.2 Influence on milk yield during the first 120 d of lactation

The results of the analysis of variance are shown in Table 5. The analysis of variance showed that breed had no influence on milk yield (in kg) during the first 120 d of the first lactation. The first kidding class had a significant influence on the milk yield in the first 120 d of lactation during the first lactation. Dairy goats that had their first kid at more than 620 d old yielded significantly more milk in the first 120 d of lactation than animals that were younger than 620 d at first kidding (+ 24.85 kg). The litter size at first kidding had a significant influence on milk yield during the first 120 d of lactation during the first lactation: if the litter size was larger than one at the first kidding, the milk yield increased by 26.33 kg during the first 120 d of lactation. Dairy goats that were selected for an extended first lactation had a significantly higher milk yield (19.72 kg more) during the first 120 d of lactation.

**Table 5:** LSM values during the first 120 d of the first lactation in dairy goats.

Factor levels during first lactation	No. obs.	Milk yield 1–120 DIM	
		LSM	SE
GF	7728	342.33${}^{a}$	7.27
GW	1464	347.93${}^{a}$	7.83
Birth year (1988–2006)	9192	${}^{***}$	
Cfk 1: ≤ 620 d	6578	332.70${}^{a}$	7.41
Cfk 2: ≥ 621 d	2614	357.55${}^{b}$	7.44
Litter size = 1	3336	330.86${}^{a}$	7.30
Litter size > 1	5571	357.19${}^{b}$	7.22
EL_1: no	7500	335.27${}^{a}$	7.28
EL_1: yes	1692	354.99${}^{b}$	7.59
No. observations	9192		

${}^{a,\;b,\;c,\;d}$ Indices within the traits show significant differences at the p<0.05 level. Production trait: milk yield during the first 120 d of lactation (DIM). Factor levels: German Fawn (GF) and German White (GW) breeds; class of first kidding age (Cfk); extended lactation during first lactation (EL_1), where “no” denotes that the first lactation was a standard lactation, and “yes” denotes that first lactation was an extended lactation. SE denotes standard error. ${}^{***}$ p<0.05.

**Table 6:** Heritability, additive genetic correlations, and phenotypic correlations between lifetime productivity traits and milk yield during the first 120 d of lactation.

Traits	LPL	LEF	EDM	Prot. cont.	Fat cont.	FPR	UC	Milk yield
				(%)	(%)			1–120 DIM
LPL	**0.22** (0.02)	0.65 (0.06)	0.29 (0.07)	0.03 (0.06)	-0.12 (0.07)	-0.25 (0.08)	-0.24 (0.08)	0.30 (0.08)
LEF	0.46 (0.03)	**0.29** (0.03)	0.81 (0.05)	-0.23 (0.06)	-0.34 (0.07)	-0.32 (0.08)	-0.17 (0.09)	0.82 (0.04)
EDM	0.08 (0.02)	0.60 (0.03)	**0.44** (0.03)	-0.37 (0.05)	-0.40 (0.06)	-0.29 (0.08)	-0.11 (0.07)	0.89 (0.03)
Prot. cont. (%)	0.01 (0.03)	-0.08 (0.03)	-0.16 (0.02)	**0.63** (0.02)	0.73 (0.03)	0.21 (0.06)	0.16 (0.06)	-0.34 (0.06)
Fat cont. (%)	0.03 (0.03)	-0.11 (0.03)	-0.12 (0.03)	0.49 (0.04)	**0.52** (0.02)	0.83 (0.02)	0.15 (0.07)	-0.25 (0.07)
FPR	0.04 (0.03)	-0.07 (0.03)	-0.03 (0.03)	-0.06 (0.04)	0.83 (0.07)	**0.32** (0.03)	0.10 (0.08)	-0.09 (0.09)
UC	0.04 (0.03)	-0.02 (0.03)	0.00 (0.02)	0.24 (0.03)	0.23 (0.03)	0.12 (0.04)	**0.47** (0.04)	-0.24 (0.09)
Milk yield	0.07 (0.02)	0.23 (0.03)	0.27 (0.02)	-0.09 (0.02)	-0.09 (0.03)	-0.05 (0.03)	-0.02 (0.03)	**0.34** (0.03)
1–120 DIM								

Heritability (on the diagonal line denoted using bold font), additive genetic correlations (above the diagonal line denoted using bold font), and phenotypic correlations (below the diagonal line denoted using bold font), with the standard errors given in parentheses. Lifetime productivity traits: length of productive life (LPL; in days), lifetime efficiency (LEF; in kg), milk yield efficiency with respect the total number of lactating days (EDM; in kg), protein content (prot. cont.; in %), fat content (fat cont.; in %), fat-to-protein ratio (FPR), urea content in milk (UC; in mg/100 mL), milk yield during the first 120 d of lactation (DIM) during the first lactation. n = 9067.

### 3.3 Estimation of variance components

The phenotypic and genetic correlations between lactation yields during the first lactation and over the animals' lifetime are presented in Table 6; the P values are shown in Table 7. The milk yield during the first lactation period had a low phenotypic correlation with LPL, LEF, and EDM of 0.07, 0.23, and 0.27 respectively. The milk yield during the first lactation had a high genetic correlation with LEF and EDM (0.82 and 0.89 respectively). With a value of 0.30, the correlation between the milk yield in the first lactation and LPL was moderately positive. The genetic correlations of LPL with LEF and EDM were positive (0.65 and 0.29 respectively). The respective genetic correlations of milk protein content (%), milk fat content (%), FPR and UC with the LEF trait were in a negative middle range, with respective values of -0.23, -0.34, -0.32 and -0.17. Heritability of lifetime productivity traits are summarized diagonally (using bold font) in Table 6; the P values are shown diagonally (using bold font) in Table 7. Heritability of LPL, LEF, and EDM was 0.22, 0.29, and 0.44 respectively. For protein and fat content (%), heritability was 0.63 and 0.52 respectively. The heritability of FPR and UC was 0.32 and 0.47 respectively. The heritability for milk yield in the first 120 d of lactation was 0.34.

**Table 7:** P values for heritability and phenotypic and genetic correlations between lifetime productivity traits and milk yield during first 120 d.

Traits	LPL	LEF	EDM	Prot. cont.	Fat cont.	FPR	UC	Milk yield
				(%)	(%)			1–120 DIM
LPL	< **0.001**	< 0.001	< 0.001	0.573	0.089	0.003	0.003	< 0.001
LEF	< 0.001	< **0.001**	< 0.001	< 0.001	< 0.001	< 0.001	0.048	< 0.001
EDM	< 0.001	< 0.001	< **0.001**	< 0.001	< 0.001	< 0.001	0.129	< 0.001
Prot. cont. (%)	0.765	0.001	< 0.001	< **0.001**	< 0.001	0.001	0.009	< 0.001
Fat cont. (%)	0.303	< 0.001	< 0.001	< 0.001	< **0.001**	< 0.001	0.028	< 0.001
FPR	0.247	0.022	0.237	0.077	< 0.001	< **0.001**	0.240	0.305
UC	0.090	0.461	0.900	< 0.001	< 0.001	0.001	< **0.001**	0.010
Milk yield	0.002	< 0.001	< 0.001	0.001	0.003	0.150	0.507	< **0.001**
1–120 DIM								

P values for heritability (on the diagonal line denoted using bold font), additive genetic correlations (above the diagonal line denoted using bold font), phenotypic correlations (below the diagonal line denoted using bold font), and lifetime productivity traits: length of productive life (LPL; in days), lifetime efficiency (LEF; in kg), milk yield efficiency with respect the total number of lactating days (EDM; in kg), protein content (prot. cont.; in %), fat content (fat cont. %; in %), fat-to-protein ratio (FPR), urea content in milk (UC; in mg/100 mL), and milk yield during first 120 d of lactation (DIM) during the first lactation. n = 9067.

## 4 Discussion

### 4.1 Data set restrictions

The data structure is rather disparate. For example, in the GF group, 89 % of farms had ≤ 50 goats, whereas 71 % of goats were kept on farms with > 50 goats. Due to a high amount of natural mating, the genetic links between different herds are low. This is a general problem in genetic studies on German goat breeds, as described by Herold et al. (2018). In addition, the present data set contains a large number of animals with unknown or only partly known ancestry (90 % of sires and 50 % of dams are missing). Zumbach et al. (2004, 2008) also encountered identification problems in their data from German goat breeding associations and could only use half of the delivered test day data for analyses. The inhomogeneous data structure and missing pedigree information can lead to an underestimation of genetic relationships within the population. Animals with unknown pedigree are assigned to phantom parents that are assumed to be unrelated. In a population with many missing records, as in our case, it is likely that relations between animals are underestimated due to missing information. Thus, these assumptions can lead to an overestimation of heritability. The standard errors in the calculations are also potentially greater, and the results can be slightly distorted and, thus, overrated or underrated. It is important to consider this background when interpreting the results. The goat population under focus is rather small; therefore, estimation of genetic parameters is difficult and still somewhat imprecise. However, for dairy goat farmers genetic gain in the goat breeding programme is important in order to increase the goats lifetime productivity and, in turn, ensure their income. The present work is a pilot study using available practical data. It can be seen as the beginning of work toward targeted breeding. Further studies will concentrate on the actual population and will include data from other breeding organizations. In the actual population, genetic relationships are better documented and the problem should be negligible.

### 4.2 Influencing factors

Class of first kidding age had a significant effect on LEF and EDM. Due to the extended rearing phase, during which an animal creates costs but does not achieve any returns, later first kidding (≥ 620 d) has a negative effect on LEF. In contrast, later kidding has a positive effect on EDM. This is similar to findings in dairy cattle, such as those by Berry and Cromie (2009), Eastham et al. (2018), and Sawa et al. (2019). In the above-mentioned studies, cows with a lower age at first calving had higher odds of survival and subsequent parities. The studies concluded that a lower age at first calving is associated with an increased lifetime daily milk yield. Depending on their internal goals, goat dairy farms must individually decide how old the animals should be at first kidding. Factors influencing the decision in this context are rearing costs, the intensity of dairy goat production on the farm, and the goal of a long productive life for the animals. If there is the potential to generate economic income from landscape conservation before the first kidding, for example, this can be an argument for later kidding (e.g. in the second year of life) if the income from landscape conservation is similar to the potential profit from milk sale during this time.

Animals with a higher milk yield during the first 120 d of lactation were more frequently chosen for extended lactation. Lehmann et al. (2017) report corresponding results for dairy cows: milk yield at the second and third milk recording during the first lactation is a good indicator of the suitability of a cow for extended lactation. Additionally, a high persistency in late lactation (around day 240–300) is a basis for the decision of whether lactation should be extended or the animal should be dried off (Herold et al., 2019). Furthermore, it was found that animals that had extended lactation of >0 % to ≤ 50 % achieved significantly higher LSM values for LPL, LEF, and EDM. These goats had short extended lactation of less than 2 years. It is likely that rebreeding was delayed for goats in this class, in order to utilize their full performance capacity. This is similar to findings on extended lactation in high-production dairy cows, where extended lactation lasts around 16 months (Sehested et al., 2019). With this strategy, extended lactation can be used to reduce offspring but still use animals with high genetic potential for reproduction.

### 4.3 Lifetime productivity traits

Data analysis showed that the milk yield during the first 120 d of lactation was phenotypically positively correlated with the lifetime productivity traits. A highly positive phenotypic correlation between the first and following lactation was also reported by Mucha et al. (2014). The milk yield at the beginning of the first lactation was positively correlated at both a phenotypic and a genetic level with LPL, LEF, and EDM. This was also confirmed in Wangler et al. (2009) for dairy cows. Milk yield is included in all of the examined traits; therefore, there is a high overlap with lifetime productivity traits. Genetic correlations between LPL, LEF, and EDM or the milk quantity during the first 120 d of lactation were in a middle to high positive range. This is favourable concerning the breeding programme, but careful consideration is required regarding which traits will be used for selection. In terms of the milk composition, the genetic correlations between LEF, EDM, and milk yield during the first 120 d of lactation, and the milk protein and fat content were – as in Boichard et al. (1989) and Torres-Vázquez et al. (2009) – in the middle negative range. This underpins the fact that protein and fat content need to be included in the selection index to ensure genetic gain in these traits. Goat milk is mainly used for cheese production, and milk solids are important components. The heritability of LPL was in the middle range at 0.24. In a study by Castañeda-Bustos et al. (2014), the estimated heritability for productive life (72 months maximum) was also in the middle range at 0.22. The heritability for LEF has previously been described in Geddes et al. (2018) using the average lifetime daily milk yield, and a value of 0.46 was estimated. In the investigated population, the heritability for LEF was lower (0.31). The highest heritability determined in Boichard et al. (1989), Torres-Vázquez et al. (2009), Rupp et al. (2011), and Castañeda-Bustos et al. (2014), as well as in the present investigation, was for lifetime protein and fat content. The heritability of the first 120 d of lactation in the present investigation was similar to the heritability of milk yield during the first lactation of 0.34 reported in Desire et al. (2018). This differs from the results of Zumbach et al. (2004, 2008) on the same German breeds. They estimated a heritability of 0.28 for milk yield from day 91 to 120 during the first lactation. They used a larger data set than the present study, 36 144 test day records for the first lactation of goats from six breeding associations, including Bavarian data. For 62 % of the studied animals, the complete pedigrees were known. As already described above, this difference might be a hint that heritability in the present study might be overestimated.

Sustainable production includes ecological and economic as well as social and ethical aspects (IFOAM, 2015). A long and productive lifetime fulfils all four criteria. An increased LPL is interesting from an economic point of view (Fekete et al., 2012; Schuster et al., 2020; de Vries and Marcondes, 2020). Increasing LPL would support the attainment of an animal's physiological peak, which, according to Bömkes et al. (2004a, b) and Gall (2001), is in the third to fourth lactation for dairy goats. Studies by Wangler et al. (2009) and Eilers (2014) showed that dairy cows also leave the herd before reaching their peak milk yield. If an animal does not reach its physiological peak due to a previous selection, this reduces the chance that it can recuperate the costs of its rearing phase, which is generally a negative from an economic point of view. Furthermore, results from a study on cattle showed that older animals seem to develop digestive and metabolic strategies to make more efficient use of nutritional energy than younger animals (Grandl et al., 2017). In terms of feed efficiency, which can affect the sustainability and profitability of a system, it would be interesting to investigate how the metabolic strategies of dairy goats change with advancing age. Additionally, a high LPL can be a component to reduce greenhouse gas emission in dairy production (Knapp et al., 2014; Grandl et al., 2019; de Vries and Marcondes, 2020). A high LPL can also be an indicator of animal welfare, of good integration in the herd, and of a high ability to cope with the farm's environmental conditions (Schuster et al., 2020; de Vries and Marcondes, 2020). Therefore, LPL also covers social and ethical aspects of sustainable dairy production.

The LEF trait essentially covers the economic aspect of lifetime productivity and implies the rearing phase, the dry phase, and the length of productive life. Rearing and dry phases that are too long have a negative effect on the LEF and also have the potential to negatively affect the profitability, as the costs of feeding and care are not balanced out by performance (Wangler et al., 2009; Eilers, 2014). Many studies on dairy cattle indicate that the length and quality of the rearing period are decisive for milk performance in the first lactation and for lifetime productivity (e.g. Le Cozler et al., 2008; Mohd Nor et al., 2013; Volkmann et al., 2019). Therefore, selection for the LEF has to consider age and body development at first insemination (Handcock et al., 2019; Volkmann et al., 2019). According to these different studies in dairy cattle, it might have negative effects on animal welfare and on profitability if animals are too young at the start of their first lactation. Extended lactation can support the LEF (Sehested et al., 2019). Different authors argue that extended lactation has a positive effect on cows' health – for example, by avoiding stressful calving and the risk of metabolic diseases (e.g. Sehested et al., 2019; Römer et al., 2020). In addition, with extended lactation, less calves or kids are born and have to be reared and marketed. This takes some of the weight off the market and off the farmers (Ringdorfer, 2009; Sehested et al., 2019).

In addition to the milk yield of the dam, milk yield during the first 120 d of the first lactation could be a reliable indicator when estimating a lifetime productivity breeding value early on. Data in the present study were limited to goats that had already left the herd. To calculate a breeding value or breeding value index, the results of the present study must be transferred to the actual population.

### 4.4 Biomarker as indicators of dairy goat health

In the present study, the average FPR values were at the lower bound of the optimum range of 1.0 to <1.5 according to Rahmann (2007). The FPR is negatively genetically correlated with lifetime productivity. This is favourable in the context of the breeding programme. The data analysis showed that the LSM of lifetime UC exceeded the limits recommended by Bellof and Weppert (1996) and von Korn et al. (2013), but they were lower than in Criscioni et al. (2016). Criscioni et al. (2016) found that the UC is higher in late lactation than in early lactation. Von Korn et al. (2013) stated that the trend in UC is more meaningful than single values. In the present study, UC is negatively genetically correlated with the lifetime productivity traits, which is favourable with respect to the breeding programme. With high lifetime productivity, UC will decrease, which will have a positive effect on animal health, the environment, and sustainability (Rensing et al., 2019). The results for the FPR and UC biomarkers are promising regarding using them as indicators of dairy goat health in the breeding programme. Both parameters are generated from routine milk recording and could serve as selection criteria for metabolic stability, as is already implemented in dairy cattle breeding (Buttchereit et al., 2011, 2012; Koeck et al., 2014; König and May, 2019). Additionally, a goat herd health data recording system was implemented to collect direct health data on farms (Herold et al., 2017), and data are slowly accumulating. In the future, when more direct health data are available and can be combined with the FPR and UC from milk recording, health traits can be incorporated into breeding value estimation and the breeding programme.

## 5 Conclusions

In summary, LPL, LEF, and EDM are suitable traits to indicate lifetime productivity in dairy goats. It is now up to the breeding associations to decide which traits should be implemented in the breeding programme. It would also be possible to create an index of different lifetime productivity traits. An additional indicator for lifetime productivity could be the milk yield during the first 120 d of the first lactation. The FPR and UC are promising indicator traits of health and robustness in dairy goats. The present study showed the importance of considering extended lactation in the breeding programme as well as modelling extended lactation in breeding value estimation. Research in dairy cattle support the fact that extended lactation can improve animal welfare and reduce the number of offspring. In dairy goats, more research is needed on the correlations between extended lactation and health and robustness traits.

## Data Availability

The data are available upon reasonable request from the corresponding author.
